# 2-[(Ferrocen-1-yl)(hy­droxy)meth­yl]prop-2-ene­nitrile

**DOI:** 10.1107/S1600536813031218

**Published:** 2013-11-20

**Authors:** S. Selvanayagam, K. Ravikumar, S. Kathiravan, R. Raghunathan

**Affiliations:** a Department of Physics, Kalasalingam University, Krishnankoil 626 126, India; b Laboratory of X-ray Crystallography, Indian Institute of Chemical Technology, Hyderabad 500 007, India; c Department of Organic Chemistry, University of Madras, Guindy Campus, Chennai 600 025, India

## Abstract

In the title ferrocene derivative, [Fe(C_5_H_5_)(C_9_H_8_NO)], the dihedral angle between the ene­nitrile group and the substituted cyclo­penta­dienyl ring is 71.2 (1)°. The cyclopentadienyl rings of the ferrocene moiety are arranged in an eclipsed conformation. The hy­droxy group, and the corresponding methine H atom, are disordered over two sets of sites with site-occupancy factors of 0.744 (4) and 0.256 (4). An intra­molecular C—H⋯O close contact is observed. In the crystal, O—H⋯N hydrogen bonds form a *C*(6) chain along [100].

## Related literature

For general background to ferrocene derivatives, see: Li *et al.* (2013 ▶); Skiba *et al.* (2012 ▶); Karolyi *et al.* (2012 ▶). For related structures, see: Leka *et al.* (2012*a* ▶,*b* ▶).
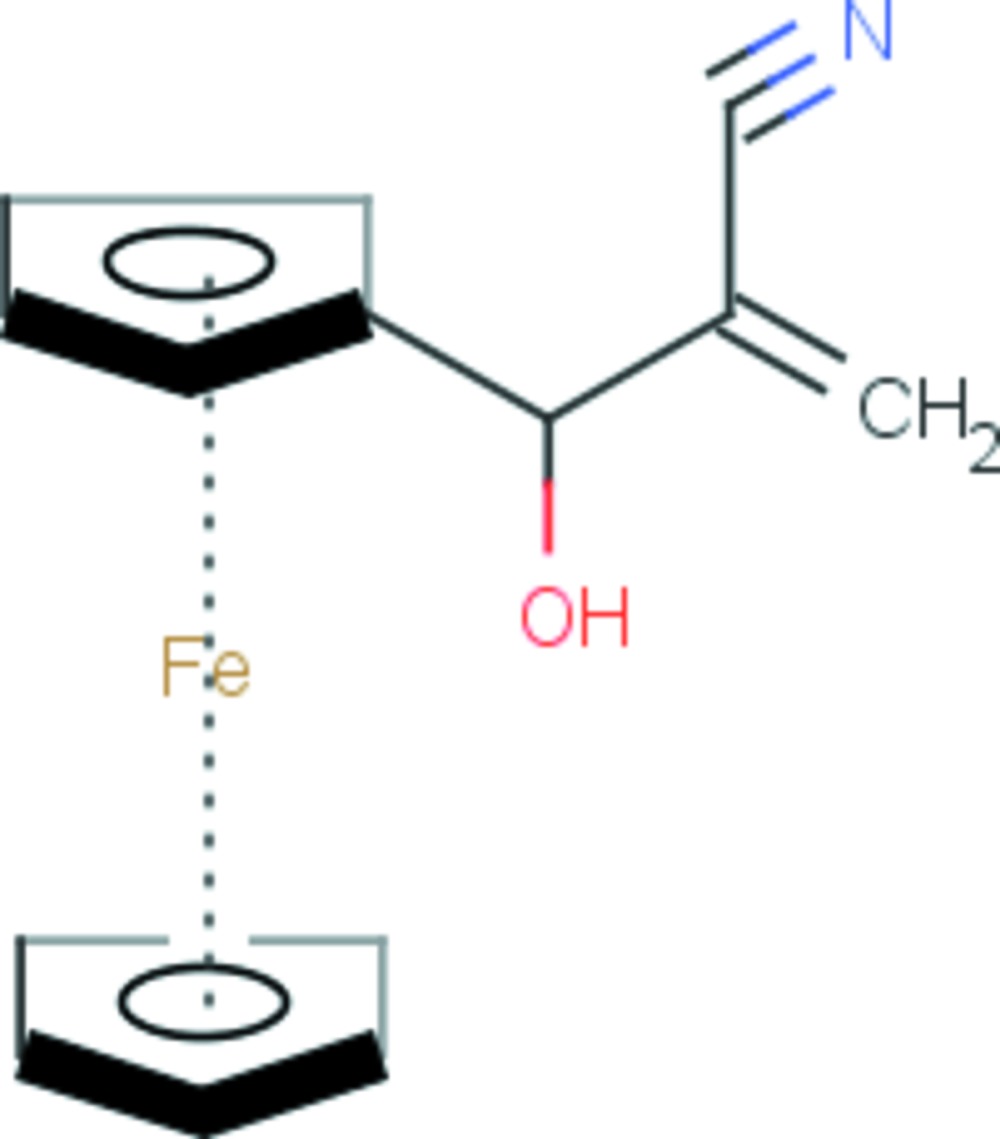


## Experimental

### 

#### Crystal data

[Fe(C_5_H_5_)(C_9_H_8_NO)]
*M* *_r_* = 267.10
Triclinic, 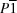

*a* = 7.4317 (15) Å
*b* = 8.0137 (16) Å
*c* = 10.083 (2) Å
α = 92.09 (3)°
β = 93.81 (3)°
γ = 101.09 (3)°
*V* = 587.3 (2) Å^3^
*Z* = 2
Mo *K*α radiation
μ = 1.26 mm^−1^
*T* = 292 K
0.22 × 0.20 × 0.18 mm

#### Data collection

Bruker SMART APEX CCD area-detector diffractometer
6351 measured reflections
2683 independent reflections
2481 reflections with *I* > 2σ(*I*)
*R*_int_ = 0.028

#### Refinement

*R*[*F* ^2^ > 2σ(*F* ^2^)] = 0.041
*wR*(*F* ^2^) = 0.112
*S* = 1.06
2683 reflections
160 parameters
3 restraints
H-atom parameters constrained
Δρ_max_ = 0.78 e Å^−3^
Δρ_min_ = −0.51 e Å^−3^

### 

Data collection: *SMART* (Bruker, 2001 ▶); cell refinement: *SAINT* (Bruker, 2001 ▶); data reduction: *SAINT*; program(s) used to solve structure: *SHELXS97* (Sheldrick, 2008 ▶); program(s) used to refine structure: *SHELXL2013* (Sheldrick, 2008 ▶); molecular graphics: *ORTEP-3 for Windows* (Farrugia, 2012 ▶) and *PLATON* (Spek, 2009 ▶); software used to prepare material for publication: *SHELXL2013* and *PLATON* (Spek, 2009 ▶).

## Supplementary Material

Crystal structure: contains datablock(s) I, global. DOI: 10.1107/S1600536813031218/zq2212sup1.cif

Structure factors: contains datablock(s) I. DOI: 10.1107/S1600536813031218/zq2212Isup2.hkl

Additional supplementary materials:  crystallographic information; 3D view; checkCIF report

## Figures and Tables

**Table 1:** Hydrogen-bond geometry (Å, °)

*D*—H⋯*A*	*D*—H	H⋯*A*	*D*⋯*A*	*D*—H⋯*A*
O1—H1⋯N1^i^	0.82	2.15	2.841 (3)	141
C14—H14*B*⋯O1	0.93	2.31	2.648 (4)	101

Symmetry code: (i) 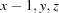.

**Fig. 1.**
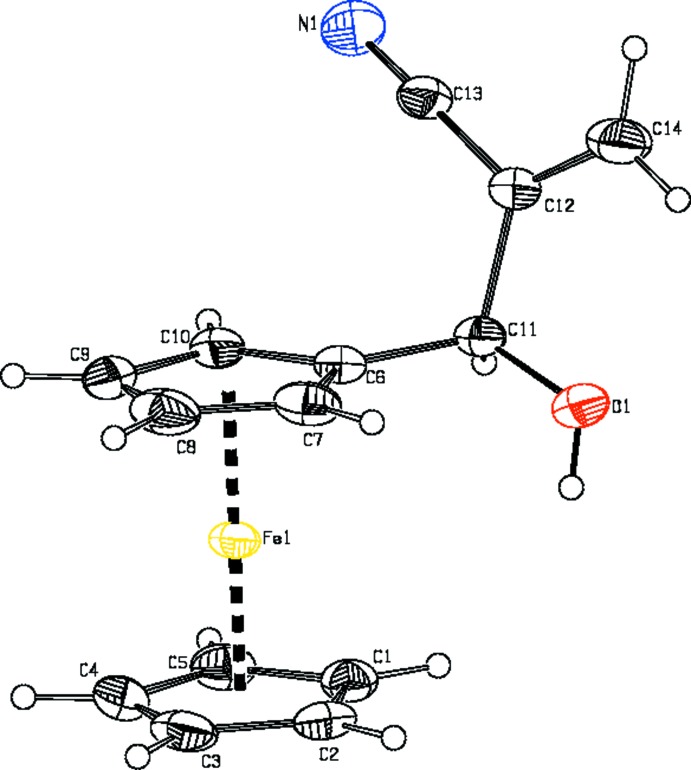
The molecular structure of the title compound, showing the atom-numbering scheme. Displacement ellipsoids are drawn at the 30% probability level. The minor occupied atoms of the disordered part have been omitted for clarity

**Fig. 2.**
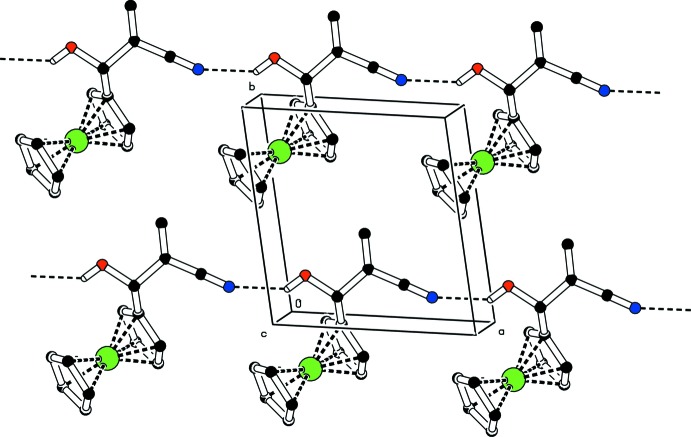
Molecular packing of the title compound, viewed along the c axis; H-bonds are shown as dashed lines. The minor occupied atoms of the disordered part have been omitted for clarity. For the sake of clarity, H atoms, not involved in hydrogen bonds, have been omitted.

## supplementary crystallographic information

### 1. Comment

Ferrocene derivatives posses antimicrobial (Li *et al.*, 2013), antibacterial (Skiba *et al.*, 2012) and antitumor (Karolyi *et al.*, 2012) activities. In view of these important properties, we have undertaken the crystal structure determination of the title compound, and the results are presented here.

The X-ray study confirmed the molecular structure and atomic connectivity of the title compound, as illustrated in Fig. 1. The geometry of the ferrocenyl group is comparable with the related literature (Leka *et al.*, 2012*a*,*b*). The cyclopentadienyl rings are almost parallel forming a dihedral angle between the mean planes of the rings of 0.18 (1)°, while the distances of Fe1 to Cg1 (C1–C5) and Cg2 (C6–C10) centroids are 1.643 (1) and 1.637 (1) Å, respectively. The bond length C13—N1 of 1.124 (3) Å confirms the triple bond character. The enenitrile group is almost perpendicular to the cyclopentadienyl ring, oriented with a dihedral angle of 71.2 (1)° with respect to the mean plane of the ring. The hydroxy group, and the corresponding methine H atom, are disordered over two orientations, with site-occupancy factors of 0.744 (4) and 0.256 (4).

In addition to the van der Waals interactions, the molecular structure is influenced by intramolecular C—H···O interactions. In the molecular packing, O-H···N hydrogen bonds form a C(6) chain motif along the *ac* plane of the unit cell (Fig. 2).

### 2. Experimental

A mixture of ferrocenecarboxaldehyde (10 mmol), acrylonitrile (12 mmol) and 1,4-diazabicyclo[2.2.2]octance (20 mol%) was stirred at room temperature for about 30 days. Silica gel was added to the reaction mixure and the product was isolated by using hexane/ethylacetate (3:1) as eluent. Single crystals of the title compound were obtained by slow evaporation of a methanol solution of the title compound at room temperature.

### 3. Refinement

H atoms were placed in idealized positions and allowed to ride on their parent atoms, with C—H distances in the range 0.93-0.98 Å, and *U*_iso_(H) = 1.2*U*_eq_(C) for H atoms. The atoms H11 and O1 are disordered over two positions: the major component exhibits a refined site-occupancy factor of 0.744 (4). The bond distances C11—O1 and C11—O1' were restrained with a DFIX command (Sheldrick, 2008) Å while the anisotropic thermal parameters of O1' and H11' were set to those of O1 and H11 [EADP instruction; Sheldrick, 2008)].

### Crystal data

**Table:**

[Fe(C_5_H_5_)(C_9_H_8_NO)]	*Z* = 2
*M**_r_* = 267.10	*F*(000) = 276
Triclinic, *P*1	*D*_x_ = 1.511 Mg m^−^^3^
*a* = 7.4317 (15) Å	Mo *K*α radiation, λ = 0.71073 Å
*b* = 8.0137 (16) Å	Cell parameters from 5048 reflections
*c* = 10.083 (2) Å	θ = 2.6–24.8°
α = 92.09 (3)°	µ = 1.26 mm^−^^1^
β = 93.81 (3)°	*T* = 292 K
γ = 101.09 (3)°	Block, colourless
*V* = 587.3 (2) Å^3^	0.22 × 0.20 × 0.18 mm

### Data collection

**Table:**

Bruker SMART APEX CCD area-detector diffractometer	*R*_int_ = 0.028
Radiation source: fine-focus sealed tube	θ_max_ = 28.1°, θ_min_ = 2.0°
ω scans	*h* = −9→9
6351 measured reflections	*k* = −10→10
2683 independent reflections	*l* = −13→13
2481 reflections with *I* > 2σ(*I*)	

### Refinement

**Table:**

Refinement on *F*^2^	3 restraints
Least-squares matrix: full	Hydrogen site location: inferred from neighbouring sites
*R*[*F*^2^ > 2σ(*F*^2^)] = 0.041	H-atom parameters constrained
*wR*(*F*^2^) = 0.112	*w* = 1/[σ^2^(*F*_o_^2^) + (0.0721*P*)^2^ + 0.143*P*] where *P* = (*F*_o_^2^ + 2*F*_c_^2^)/3
*S* = 1.06	(Δ/σ)_max_ = 0.001
2683 reflections	Δρ_max_ = 0.78 e Å^−^^3^
160 parameters	Δρ_min_ = −0.51 e Å^−^^3^

### Special details

**Table:**

Geometry. All esds (except the esd in the dihedral angle between two l.s. planes) are estimated using the full covariance matrix. The cell esds are taken into account individually in the estimation of esds in distances, angles and torsion angles; correlations between esds in cell parameters are only used when they are defined by crystal symmetry. An approximate (isotropic) treatment of cell esds is used for estimating esds involving l.s. planes.

### Fractional atomic coordinates and isotropic or equivalent isotropic displacement parameters (Å^2^)

**Table:**

	*x*	*y*	*z*	*U*_iso_*/*U*_eq_	Occ. (<1)
Fe1	0.12344 (3)	0.78280 (3)	0.68844 (3)	0.03652 (14)	
O1	0.1780 (3)	1.2232 (3)	0.8242 (3)	0.0630 (7)	0.744 (4)
H1	0.0808	1.1576	0.8334	0.094*	0.744 (4)
O1'	0.3410 (9)	1.0729 (9)	0.9630 (6)	0.0630 (7)	0.256 (4)
H1'	0.4204	1.0145	0.9653	0.094*	0.256 (4)
N1	0.7948 (3)	1.1554 (3)	0.8583 (3)	0.0685 (7)	
C1	−0.0590 (3)	0.7684 (3)	0.8309 (2)	0.0469 (5)	
H1A	−0.0533	0.8494	0.9070	0.056*	
C2	−0.1490 (3)	0.7765 (3)	0.7047 (3)	0.0495 (5)	
H2	−0.2169	0.8643	0.6770	0.059*	
C3	−0.1239 (4)	0.6360 (4)	0.6253 (3)	0.0580 (7)	
H3	−0.1711	0.6094	0.5323	0.070*	
C4	−0.0192 (4)	0.5410 (3)	0.7013 (3)	0.0592 (7)	
H4	0.0191	0.4364	0.6713	0.071*	
C5	0.0208 (4)	0.6227 (3)	0.8283 (3)	0.0529 (6)	
H5	0.0925	0.5850	0.9025	0.063*	
C6	0.3135 (3)	0.9999 (3)	0.7279 (2)	0.0370 (4)	
C7	0.2271 (3)	0.9998 (3)	0.5987 (2)	0.0500 (6)	
H7	0.1574	1.0840	0.5659	0.060*	
C8	0.2586 (4)	0.8557 (4)	0.5254 (3)	0.0637 (8)	
H8	0.2132	0.8225	0.4329	0.076*	
C9	0.3633 (4)	0.7675 (4)	0.6070 (3)	0.0597 (7)	
H9	0.4043	0.6625	0.5817	0.072*	
C10	0.3985 (3)	0.8559 (3)	0.7331 (3)	0.0467 (5)	
H10	0.4685	0.8232	0.8104	0.056*	
C11	0.3231 (3)	1.1352 (3)	0.8366 (2)	0.0400 (4)	
H11	0.3218	1.0822	0.9227	0.048*	0.744 (4)
H11'	0.2147	1.1880	0.8276	0.048*	0.256 (4)
C12	0.4964 (3)	1.2685 (3)	0.8351 (2)	0.0438 (5)	
C13	0.6643 (3)	1.2065 (3)	0.8478 (2)	0.0464 (5)	
C14	0.5015 (4)	1.4309 (4)	0.8248 (4)	0.0779 (10)	
H14A	0.6141	1.5058	0.8257	0.093*	
H14B	0.3927	1.4719	0.8165	0.093*	

### Atomic displacement parameters (Å^2^)

**Table:**

	*U*^11^	*U*^22^	*U*^33^	*U*^12^	*U*^13^	*U*^23^
Fe1	0.02602 (18)	0.0411 (2)	0.03825 (19)	−0.00367 (12)	0.00291 (12)	0.00018 (12)
O1	0.0324 (11)	0.0528 (13)	0.103 (2)	0.0050 (9)	0.0150 (11)	−0.0021 (12)
O1'	0.0324 (11)	0.0528 (13)	0.103 (2)	0.0050 (9)	0.0150 (11)	−0.0021 (12)
N1	0.0404 (12)	0.0762 (16)	0.0859 (17)	0.0112 (11)	−0.0096 (11)	−0.0092 (13)
C1	0.0379 (11)	0.0524 (13)	0.0459 (12)	−0.0047 (10)	0.0120 (9)	0.0006 (9)
C2	0.0257 (10)	0.0587 (14)	0.0610 (14)	−0.0013 (9)	0.0059 (9)	0.0095 (11)
C3	0.0390 (12)	0.0699 (17)	0.0520 (13)	−0.0194 (11)	0.0021 (10)	−0.0081 (12)
C4	0.0482 (14)	0.0414 (12)	0.0826 (18)	−0.0087 (10)	0.0201 (13)	−0.0034 (12)
C5	0.0424 (12)	0.0532 (14)	0.0602 (14)	−0.0018 (10)	0.0081 (10)	0.0183 (11)
C6	0.0262 (9)	0.0397 (10)	0.0420 (10)	−0.0015 (7)	0.0018 (7)	0.0044 (8)
C7	0.0408 (12)	0.0600 (14)	0.0424 (11)	−0.0091 (10)	0.0014 (9)	0.0140 (10)
C8	0.0546 (16)	0.0820 (19)	0.0410 (12)	−0.0224 (14)	0.0153 (11)	−0.0083 (12)
C9	0.0375 (12)	0.0615 (16)	0.0745 (18)	−0.0049 (11)	0.0198 (12)	−0.0197 (13)
C10	0.0261 (9)	0.0467 (12)	0.0640 (14)	0.0007 (8)	0.0004 (9)	−0.0024 (10)
C11	0.0301 (10)	0.0391 (10)	0.0482 (11)	−0.0006 (8)	0.0059 (8)	0.0035 (8)
C12	0.0311 (10)	0.0427 (11)	0.0547 (12)	0.0002 (8)	0.0035 (9)	−0.0022 (9)
C13	0.0321 (11)	0.0499 (12)	0.0521 (12)	−0.0022 (9)	−0.0008 (9)	−0.0052 (9)
C14	0.0422 (14)	0.0460 (15)	0.143 (3)	−0.0022 (12)	0.0181 (17)	0.0090 (17)

### Geometric parameters (Å, º)

**Table:**

Fe1—C6	2.027 (2)	C3—H3	0.9800
Fe1—C5	2.029 (2)	C4—C5	1.404 (4)
Fe1—C8	2.029 (3)	C4—H4	0.9800
Fe1—C10	2.030 (2)	C5—H5	0.9800
Fe1—C1	2.031 (2)	C6—C7	1.414 (3)
Fe1—C3	2.032 (2)	C6—C10	1.419 (3)
Fe1—C7	2.032 (2)	C6—C11	1.501 (3)
Fe1—C2	2.033 (2)	C7—C8	1.413 (4)
Fe1—C9	2.034 (3)	C7—H7	0.9800
Fe1—C4	2.035 (3)	C8—C9	1.397 (5)
O1—C11	1.399 (3)	C8—H8	0.9800
O1—H1	0.8200	C9—C10	1.416 (4)
O1'—C11	1.394 (6)	C9—H9	0.9800
O1'—H1'	0.8200	C10—H10	0.9800
N1—C13	1.124 (3)	C11—C12	1.508 (3)
C1—C2	1.408 (4)	C11—H11	0.9800
C1—C5	1.408 (4)	C11—H11'	0.9800
C1—H1A	0.9800	C12—C14	1.303 (4)
C2—C3	1.406 (4)	C12—C13	1.429 (3)
C2—H2	0.9800	C14—H14A	0.9300
C3—C4	1.402 (4)	C14—H14B	0.9300
			
C6—Fe1—C5	124.47 (10)	C2—C3—H3	125.8
C6—Fe1—C8	68.46 (10)	Fe1—C3—H3	125.8
C5—Fe1—C8	156.88 (14)	C3—C4—C5	107.7 (2)
C6—Fe1—C10	40.94 (9)	C3—C4—Fe1	69.70 (15)
C5—Fe1—C10	107.84 (11)	C5—C4—Fe1	69.54 (15)
C8—Fe1—C10	68.20 (12)	C3—C4—H4	126.2
C6—Fe1—C1	107.63 (10)	C5—C4—H4	126.2
C5—Fe1—C1	40.59 (11)	Fe1—C4—H4	126.2
C8—Fe1—C1	161.02 (14)	C4—C5—C1	108.4 (2)
C10—Fe1—C1	121.84 (11)	C4—C5—Fe1	70.04 (15)
C6—Fe1—C3	157.18 (12)	C1—C5—Fe1	69.80 (14)
C5—Fe1—C3	67.81 (12)	C4—C5—H5	125.8
C8—Fe1—C3	107.91 (11)	C1—C5—H5	125.8
C10—Fe1—C3	160.59 (12)	Fe1—C5—H5	125.8
C1—Fe1—C3	68.06 (11)	C7—C6—C10	107.8 (2)
C6—Fe1—C7	40.76 (9)	C7—C6—C11	125.7 (2)
C5—Fe1—C7	161.10 (12)	C10—C6—C11	126.4 (2)
C8—Fe1—C7	40.73 (12)	C7—C6—Fe1	69.79 (13)
C10—Fe1—C7	68.59 (11)	C10—C6—Fe1	69.64 (12)
C1—Fe1—C7	124.28 (11)	C11—C6—Fe1	128.96 (15)
C3—Fe1—C7	121.75 (11)	C8—C7—C6	107.6 (2)
C6—Fe1—C2	121.64 (10)	C8—C7—Fe1	69.52 (15)
C5—Fe1—C2	68.07 (11)	C6—C7—Fe1	69.45 (13)
C8—Fe1—C2	124.44 (13)	C8—C7—H7	126.2
C10—Fe1—C2	157.38 (11)	C6—C7—H7	126.2
C1—Fe1—C2	40.53 (10)	Fe1—C7—H7	126.2
C3—Fe1—C2	40.49 (11)	C9—C8—C7	108.8 (2)
C7—Fe1—C2	107.66 (11)	C9—C8—Fe1	70.09 (15)
C6—Fe1—C9	68.68 (10)	C7—C8—Fe1	69.75 (14)
C5—Fe1—C9	121.88 (13)	C9—C8—H8	125.6
C8—Fe1—C9	40.22 (13)	C7—C8—H8	125.6
C10—Fe1—C9	40.79 (10)	Fe1—C8—H8	125.6
C1—Fe1—C9	157.44 (13)	C8—C9—C10	108.0 (2)
C3—Fe1—C9	124.04 (11)	C8—C9—Fe1	69.69 (16)
C7—Fe1—C9	68.38 (12)	C10—C9—Fe1	69.46 (14)
C2—Fe1—C9	160.49 (12)	C8—C9—H9	126.0
C6—Fe1—C4	160.96 (12)	C10—C9—H9	126.0
C5—Fe1—C4	40.42 (12)	Fe1—C9—H9	126.0
C8—Fe1—C4	121.56 (13)	C9—C10—C6	107.8 (2)
C10—Fe1—C4	124.15 (12)	C9—C10—Fe1	69.75 (14)
C1—Fe1—C4	68.23 (11)	C6—C10—Fe1	69.42 (12)
C3—Fe1—C4	40.33 (12)	C9—C10—H10	126.1
C7—Fe1—C4	156.93 (13)	C6—C10—H10	126.1
C2—Fe1—C4	68.13 (12)	Fe1—C10—H10	126.1
C9—Fe1—C4	107.59 (12)	O1'—C11—C6	112.5 (4)
C11—O1—H1	109.5	O1—C11—C6	113.0 (2)
C11—O1'—H1'	109.5	O1'—C11—C12	102.2 (3)
C2—C1—C5	107.7 (2)	O1—C11—C12	105.74 (19)
C2—C1—Fe1	69.80 (13)	C6—C11—C12	111.08 (18)
C5—C1—Fe1	69.61 (14)	O1—C11—H11	109.0
C2—C1—H1A	126.2	C6—C11—H11	109.0
C5—C1—H1A	126.2	C12—C11—H11	109.0
Fe1—C1—H1A	126.2	O1'—C11—H11'	110.3
C3—C2—C1	107.8 (2)	C6—C11—H11'	110.3
C3—C2—Fe1	69.71 (15)	C12—C11—H11'	110.3
C1—C2—Fe1	69.67 (14)	C14—C12—C13	119.6 (2)
C3—C2—H2	126.1	C14—C12—C11	124.9 (2)
C1—C2—H2	126.1	C13—C12—C11	115.5 (2)
Fe1—C2—H2	126.1	N1—C13—C12	178.9 (3)
C4—C3—C2	108.5 (2)	C12—C14—H14A	120.0
C4—C3—Fe1	69.98 (15)	C12—C14—H14B	120.0
C2—C3—Fe1	69.81 (14)	H14A—C14—H14B	120.0
C4—C3—H3	125.8		
			
C5—C1—C2—C3	0.0 (3)	C7—C8—C9—Fe1	59.20 (18)
Fe1—C1—C2—C3	59.52 (17)	C8—C9—C10—C6	0.1 (3)
C5—C1—C2—Fe1	−59.55 (17)	Fe1—C9—C10—C6	−59.20 (16)
C1—C2—C3—C4	0.0 (3)	C8—C9—C10—Fe1	59.25 (18)
Fe1—C2—C3—C4	59.50 (18)	C7—C6—C10—C9	−0.2 (3)
C1—C2—C3—Fe1	−59.49 (16)	C11—C6—C10—C9	−176.6 (2)
C2—C3—C4—C5	0.0 (3)	Fe1—C6—C10—C9	59.40 (17)
Fe1—C3—C4—C5	59.42 (18)	C7—C6—C10—Fe1	−59.58 (16)
C2—C3—C4—Fe1	−59.39 (17)	C11—C6—C10—Fe1	124.0 (2)
C3—C4—C5—C1	0.0 (3)	C7—C6—C11—O1'	153.7 (4)
Fe1—C4—C5—C1	59.47 (17)	C10—C6—C11—O1'	−30.5 (4)
C3—C4—C5—Fe1	−59.51 (18)	Fe1—C6—C11—O1'	61.8 (4)
C2—C1—C5—C4	0.0 (3)	C7—C6—C11—O1	26.2 (3)
Fe1—C1—C5—C4	−59.62 (18)	C10—C6—C11—O1	−158.0 (2)
C2—C1—C5—Fe1	59.66 (16)	Fe1—C6—C11—O1	−65.7 (3)
C10—C6—C7—C8	0.2 (3)	C7—C6—C11—C12	−92.4 (2)
C11—C6—C7—C8	176.7 (2)	C10—C6—C11—C12	83.3 (3)
Fe1—C6—C7—C8	−59.26 (17)	Fe1—C6—C11—C12	175.68 (15)
C10—C6—C7—Fe1	59.49 (15)	O1'—C11—C12—C14	−116.6 (4)
C11—C6—C7—Fe1	−124.1 (2)	O1—C11—C12—C14	0.3 (4)
C6—C7—C8—C9	−0.2 (3)	C6—C11—C12—C14	123.2 (3)
Fe1—C7—C8—C9	−59.41 (19)	O1'—C11—C12—C13	62.6 (4)
C6—C7—C8—Fe1	59.21 (16)	O1—C11—C12—C13	179.5 (2)
C7—C8—C9—C10	0.1 (3)	C6—C11—C12—C13	−57.5 (3)
Fe1—C8—C9—C10	−59.11 (18)		

### Hydrogen-bond geometry (Å, º)

**Table:**

*D*—H···*A*	*D*—H	H···*A*	*D*···*A*	*D*—H···*A*
O1—H1···N1^i^	0.82	2.15	2.841 (3)	141
C14—H14*B*···O1	0.93	2.31	2.648 (4)	101

Symmetry code: (i) *x*−1, *y*, *z*.
